# Predicting rainfall using machine learning, deep learning, and time series models across an altitudinal gradient in the North-Western Himalayas

**DOI:** 10.1038/s41598-024-77687-x

**Published:** 2024-11-13

**Authors:** Owais Ali Wani, Syed Sheraz Mahdi, Md. Yeasin, Shamal Shasang Kumar, Alexandre S. Gagnon, Faizan Danish, Nadhir Al-Ansari, Salah El‑Hendawy, Mohamed A. Mattar

**Affiliations:** 1 Division of Agronomy, Faculty of Agriculture Wadoora, Sher-e-Kashmir University of Agricultural Sciences & Technology of Kashmir (SKUAST-K), Jammu and Kashmir, 193201 India; 2 ICAR-Indian Agricultural Statistics Research Institute, New Delhi, 110 012 India; 3 Department of Agronomy (Rootcrops), Ministry of Agriculture & Waterways (MOA & W), Suva City, 679 Fiji; 4 School of Biological and Environmental Sciences, Liverpool John Moores University, Liverpool, L3 3AF UK; 5 Department of Mathematics, School of Advanced Sciences, VIT-AP University, Inavolu, Andhra Pradesh 522237 India; 6 Civil, Environmental and Natural Resources Engineering, Lulea University of Technology, 97187 Lulea, Sweden; 7 Department of Plant Production, College of Food and Agricultural Sciences, King Saud University, P.O. Box 2460, Riyadh 11451, Saudi Arabia; 8 Department of Agricultural Engineering, College of Food and Agriculture Sciences, King Saud University, P.O. Box 2460, Riyadh 11451, Saudi Arabia; 9 Advanced Centre for Rainfed Agriculture (ACRA), Dhiansar, Bari-Brahmana-181133, SKUAST-Jammu, UT-J&K, India

**Keywords:** Hydrology, Hydrology

## Abstract

Predicting rainfall is a challenging and critical task due to its significant impact on society. Timely and accurate predictions are essential for minimizing human and financial losses. The dependence of approximately 60% of agricultural land in India on monsoon rainfall implies the crucial nature of accurate rainfall prediction. Precise rainfall forecasts can facilitate early preparedness for disasters associated with heavy rains, enabling the public and government to take necessary precautions. In the North-Western Himalayas, where meteorological data are limited, the need for improved accuracy in traditional modeling methods for rainfall forecasting is pressing. To address this, our study proposes the application of advanced machine learning (ML) algorithms, including random forest (RF), support vector regression (SVR), artificial neural network (ANN), and k-nearest neighbour (KNN) along with various deep learning (DL) algorithms such as long short-term memory (LSTM), bi-directional LSTM, deep LSTM, gated recurrent unit (GRU), and simple recurrent neural network (RNN). These advanced techniques hold the potential to significantly improve the accuracy of rainfall prediction, offering hope for more reliable forecasts. Additionally, time series techniques, including autoregressive integrated moving average (ARIMA) and trigonometric, Box-Cox transform, arma errors, trend, and seasonal components (TBATS), are proposed for predicting rainfall across the altitudinal gradients of India’s North-Western Himalayas. This approach can potentially revolutionise how we approach rainfall forecasting, ushering in a new era of accuracy and reliability. The effectiveness and accuracy of the proposed algorithms were assessed using meteorological data obtained from six weather stations at different elevations spanning from 1980 to 2021. The results indicate that DL methods exhibit the highest accuracy in predicting rainfall, as measured by the root mean squared error (RMSE) and mean absolute error (MAE), followed by ML algorithms and time series techniques. Among the DL algorithms, the accuracy order was bi-directional LSTM, LSTM, RNN, deep LSTM, and GRU. For the ML algorithms, the accuracy order was ANN, KNN, SVR, and RF. These findings suggest that altitude significantly affects the accuracy of the models, highlighting the need for additional weather stations in this mountainous region to enhance the precision of rainfall prediction.

**Supplementary Information:**

The online version contains supplementary material available at 10.1038/s41598-024-77687-x.

## Introduction

Climate change implications on food security are significantly connected to its profound impact on agriculture. Anticipating the conditions for the upcoming planting season becomes challenging due to the prevailing uncertainty caused by the unpredictable nature of climate variability, often proving detrimental to agricultural activities^1^. As a result, farmers and farming decision-makers heavily rely on their understanding of regional climatic patterns when making crucial decisions about ploughing, seeding, and managing their crops. However, traditional approaches have become less reliable in the changing climate^2^. Enhanced climate predictions offer hope for improving decision-making in the agricultural sector. These advanced forecasts have the potential to mitigate the adverse effects of factors such as a poor or delayed monsoon season and provide an opportunity to leverage projected favourable weather conditions^3^. Embracing these predictions can help farmers and other agricultural professionals navigate uncertainty more effectively, safeguarding their crops and yields while laying the groundwork for sustainable farming practices that adapt to climate change^4^. Additionally, heavy precipitation can lead to flooding, impacting infrastructure, transport networks, and human livelihood^5^. Therefore, it would be advantageous to the decision-making process if the potential magnitude of rainfall over a region could be quantified in advance. Predicting rainfall is crucial for improving agricultural output and ensuring a country’s residents’ access to food and clean water^6^.

The link between climate predictions and agriculture highlights the crucial role of accurate forecasts in shaping the future of food security. In recent decades, improving rainfall forecasting has been a focal point in the scientific community^7^. To improve the accuracy of rainfall forecasts, it is essential to integrate various data sources and utilise advanced modeling techniques. Numerical weather prediction models utilise mathematical equations to simulate the atmosphere’s behaviour and interactions with various climatic factors. These models incorporate historical data, real-time observations from weather stations, satellite imagery, and sophisticated computational algorithms^8^. Rainfall is intricately linked to a network of climatic factors, each influencing and being influenced by others. These factors include maximum and minimum temperatures, atmospheric pressure, relative humidity, and wind speed^9^. The interconnectedness of these variables creates a complex system that plays a crucial role in maintaining overall climate balance. Numerical weather prediction models aim to provide more precise forecasts by examining the intricate relationships between climatic elements and rainfall. The intricate and ever-changing nature of the atmosphere and the complex interplay of various climatic factors have challenged the improvement of rainfall forecasting. Although there has been notable progress in enhancing rainfall prediction techniques, several factors continue to contribute to the complexity of the task. One significant obstacle is the inherent unpredictability of atmospheric processes^10^. The atmosphere is dynamic and chaotic, with sudden shifts and unexpected interactions. This unpredictability suggests that even minor variations in initial conditions can lead to vastly different outcomes over time^11^. This phenomenon presents a substantial challenge for meteorologists and climate scientists working to create accurate models for predicting rainfall patterns. Another crucial aspect complicating rainfall forecasting is the need for long-term historical data to construct reliable prediction models. Historical data aids scientists in identifying rainfall patterns, trends, and cycles. However, obtaining comprehensive and high-quality historical data is not always straightforward, particularly in areas with limited monitoring infrastructure^12^.

Rainfall prediction involves complex stochastic and nonlinear behaviours, which can be addressed using advanced techniques such as data mining, artificial intelligence (AI), ML, and DL. ML methods can reveal hidden patterns in historical rainfall data and have been proposed as an alternative modeling approach for nonlinear and dynamic systems^5^. For this reason, in recent years, ML approaches have emerged as powerful successors to traditional data mining techniques in the domain of rainfall prediction, reflecting the growing recognition of ML methods’ capabilities in tackling the intricate challenges of predicting precipitation patterns^13–15^,^16^. demonstrated that ML methods are superior to traditional deterministic methods for rainfall prediction. Examples of ML models include RF, SVR, and SVM. RF employs an ensemble of decision trees to make predictions, enhancing accuracy and handling complex data relationships^17^. SVR, a form of SVM adapted for regression tasks, effectively captures nonlinear patterns in data. SVM is a versatile model for classification and regression, creating optimal decision boundaries through support vectors^18^.

DL, as a subset of ML, has also demonstrated significant potential in enhancing predictive capabilities by utilising sophisticated neural networks inspired by the interconnected neurons of the human brain^19^. DL techniques encompass various algorithms of ANNs. These networks consist of interconnected layers of nodes, or “neurons,” each processing and transforming input data before passing it to the next layer. The depth and intricacy of these networks enable them to capture complex patterns and relationships within extensive datasets. This capability is particularly valuable in domains characterised by high-dimensional and nonlinear data, such as climate science and meteorology^20^. DL methods are closely related to traditional ML methods, albeit differing in architectural complexity and the hierarchy of feature extraction^21^. While both DL and traditional ML strive to identify patterns in data, DL models excel in autonomously learning data representations at multiple levels of abstraction. This implies that DL models can automatically discover intricate features within raw data without explicit feature engineering, which often demands domain expertise and can be time-consuming^22^. DL application in rainfall prediction involves training neural networks on historical climate data. These networks are designed to identify hidden correlations, nonlinear relationships, and temporal dependencies among crucial variables for accurate rainfall forecasts^23^. As the neural networks process and learn from these datasets, they refine their internal representations, progressively enabling them to make increasingly accurate predictions. DL methods effectively harness the interconnectedness of climatic factors in rainfall forecasting, considering diverse variables such as temperature, humidity, wind speed, and atmospheric pressure and recognising their combined impact on rainfall patterns^24^. This comprehensive approach provides an advantage over traditional methods that may struggle to capture the intricate interactions among these variables. Furthermore, DL’s ability to process vast amounts of data aligns well with the requirements of meteorological forecasting, where historical climate records span many decades and comprise an array of variables. The capacity of DL models to identify subtle trends, nonlinear dependencies, and intricate temporal patterns within these datasets can lead to more accurate and reliable rainfall predictions^25^.

Examples of DL approaches include ANN, RNN, KNN, and GRU. ANN simulates interconnected neurons to capture complex relationships in data, enhancing learning capabilities. RNN specialises in sequence modeling, preserving the memory of previous inputs for tasks like language processing^26^. KNN is a learning algorithm that makes predictions based on the proximity of data points in the feature space. GRU, a variant of RNN, addresses vanishing gradient issues in RNN and improves long-range dependency capture^27^. Additionally, there are time series models such as ARIMA, LSTM, trigonometric, Box-Cox transform, ARMA, and TBATS models. These models allow data analysts to model and forecast time series data across various applications. For instance, ARIMA combines autoregressive and moving average components with differencing for handling non-stationary data. LSTM is an RNN that captures intricate temporal relationships in sequences^28,29^. Trigonometric models leverage sinusoidal functions to capture cyclic patterns, which are ideal for data with periodic fluctuations. The Box-Cox transform stabilises variance and enhances normality in data^30^. ARMA models blend autoregressive and moving average components, while TBATS considers complex trends and seasonal patterns^17^. Using linear regression as an ML approach aims to predict rainfall by establishing relationships with other atmospheric variables^31,32^^33^. conducted a thorough comparative study to assess the effectiveness of statistical modeling and regression techniques in predicting rainfall using environmental features. The study highlighted the superior performance of regression techniques over statistical modeling when forecasting rainfall patterns. Additionally, their investigation demonstrated that the RF model exhibited enhanced predictive accuracy among ML algorithms compared to SVM and Decision Tree methods. This study contributes valuable insights into rainfall prediction, shedding light on the advantages of specific modeling approaches. The DL LSTM model has proven to be effective for rainfall prediction. In a study on forecasting rain in the Hyderabad region of India^20,34^, put forth an enhanced LSTM model, which they compared with other models such as Holt-Winters, ARIMA, extreme learning machine (ELM), and RNN. The results were verified using ANN to predict the monthly average rainfall^35^. The findings indicate that out of the three different types of networks (layer recurrent, cascaded feed forward back propagation, and feed forward back propagation), the feed forward back propagation network type yielded the best results.

Previous studies have utilised a linear regression model to pinpoint crucial characteristics for predicting rainfall, including solar radiation, detectable water vapour, and daily patterns^36^. found that temperature, wind, and cyclones can be utilised to forecast rain in growth of agriculture sector and the farmers can take their decisions accordingly. Some researchers have also utilised atmospheric features such as temperature, relative humidity, pressure, and wind speed to accurately predict rainfall using ML techniques such as ANN, RF, and multiple linear regression models^37,38^. Therefore, the combination of ML, DL, and time series modeling represents a promising frontier for advancing our understanding of climatic patterns and improving our ability to predict rainfall.

This study focuses on exploring rainfall data in the North-Western Himalayas with several key objectives. Firstly, we aim to gauge the efficacy of advanced ML algorithms in predicting rainfall patterns. Secondly, we seek to evaluate the accuracy of DL models specifically tailored for this geographical region. Thirdly, we intend to compare the performance of ML and DL methods against traditional time series techniques commonly used in meteorological predictions. Additionally, our study aims to analyze how variations in altitude affect the precision of these predictive models. Lastly, based on our findings, we aim to propose a holistic approach to enhance the overall accuracy of rainfall prediction in the region. Through these objectives, we aim to contribute valuable insights that could advance both scientific understanding and practical applications in meteorological forecasting in mountainous terrains. This study takes a multidimensional approach to unravel the complexities of precipitation trends in a region known for its intricate altitude-dependent climate variations. By integrating these innovative methodologies, we aim to decode the intricate relationships between environmental factors and rainfall, ultimately developing more accurate and localised predictive models.

## Materials and methods

**Fig. 1:**
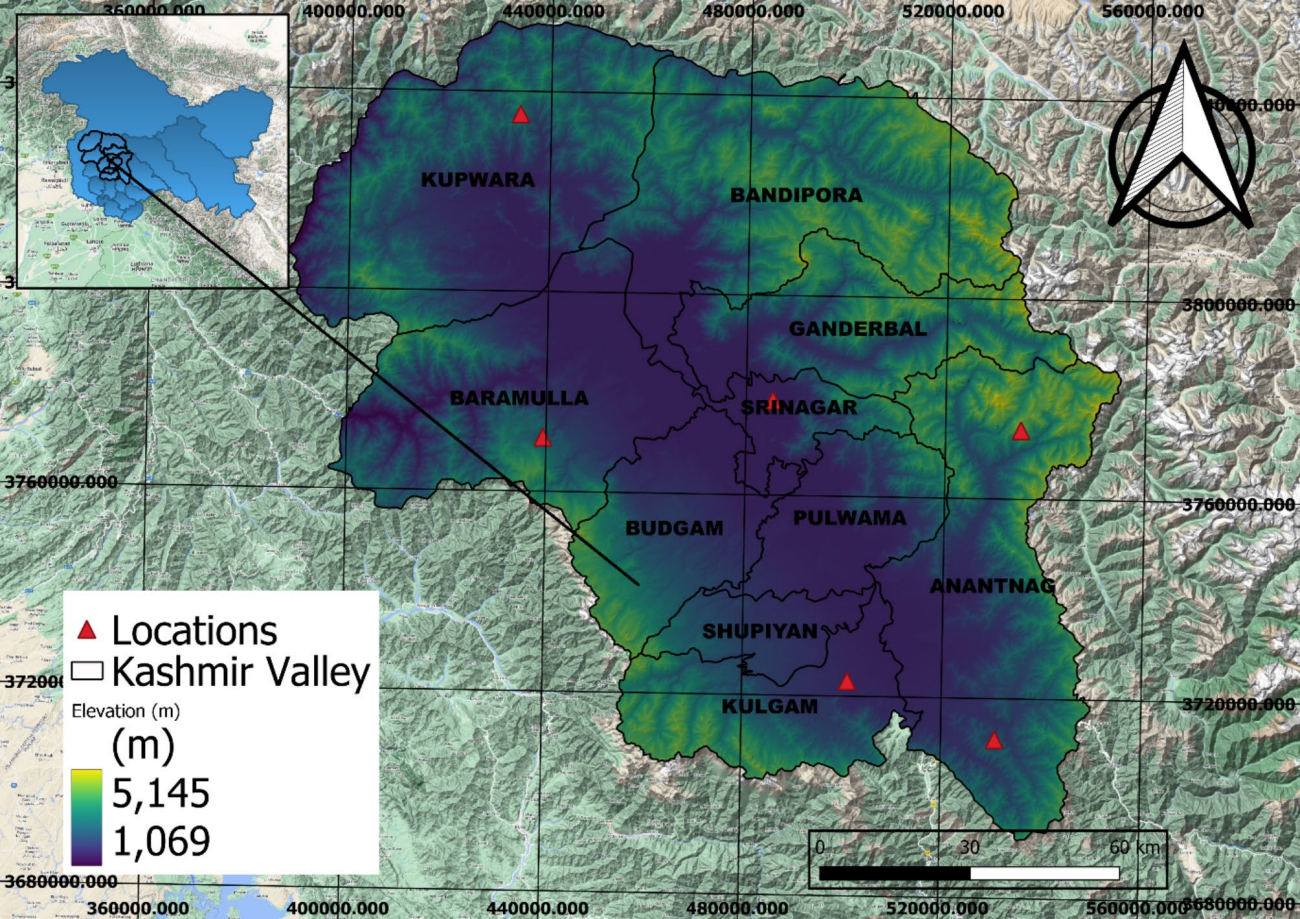
Study area map (map was created using QGIS 3.30.0 https://www.qgis.org/).

Case study region, meteorological data and pre-processing. The study area covers the North-Western Himalayan region of India, centered at approximately 33° 5′ 24′′ N latitude and 74° 47′ 24′′ E longitude. Figure 1 illustrates the location of the study area covered. The study map was generated by QGIS 3.30.0. The six locations of meteorological stations were located on map across the altitudinal gradient. The primary data was gathered from the head office of the Indian Meteorological Department (IMD). The dataset encompassed daily data for maximum and minimum temperature and rainfall spanning 40 years (1980–2020). The initial data pre-processing stage involved several tasks: data conversion, handling missing values, encoding categorical variables, and partitioning the dataset into training and testing sets. Since the raw data exhibited both missing values and extreme values or outliers, corrective actions were taken. Missing values were replaced by not available (N.A). Subsequently, the dataset underwent encoding procedures before being ready for experimentation. Relevant attributes crucial for rainfall prediction were identified and isolated. The dataset was then divided, with 80% designated for training and 20% for testing, serving as input for the models.

### Models

 This study centres on rainfall prediction through a combined approach involving ML, DL, and time series methodologies. The analysis encompassed three distinct ML algorithms: ANN, SVR, and RF; three DL algorithms: RNN, LSTM, and GRU; and two time series algorithms: ARIMA and TBATS. These various algorithms utilised input variables moderately to strongly associated with rainfall, drawn from multiple environmental factors.The model parameters and information about models is given in supplementary Table 1S. The study determined and reported the most effective models and algorithms by assessing performance using the RMSE and MAE metrics, other accuracy assement like Bias and R2 of various models across the altitudes is given in Table 1S.

### Machine learning (ML) algorithms

 Three ML algorithms were utilized to predict the rainfall based on the behaviour of the data set. The algorithms are discussed below:

### Artificial neural network (ANN)

**Fig. 2:**
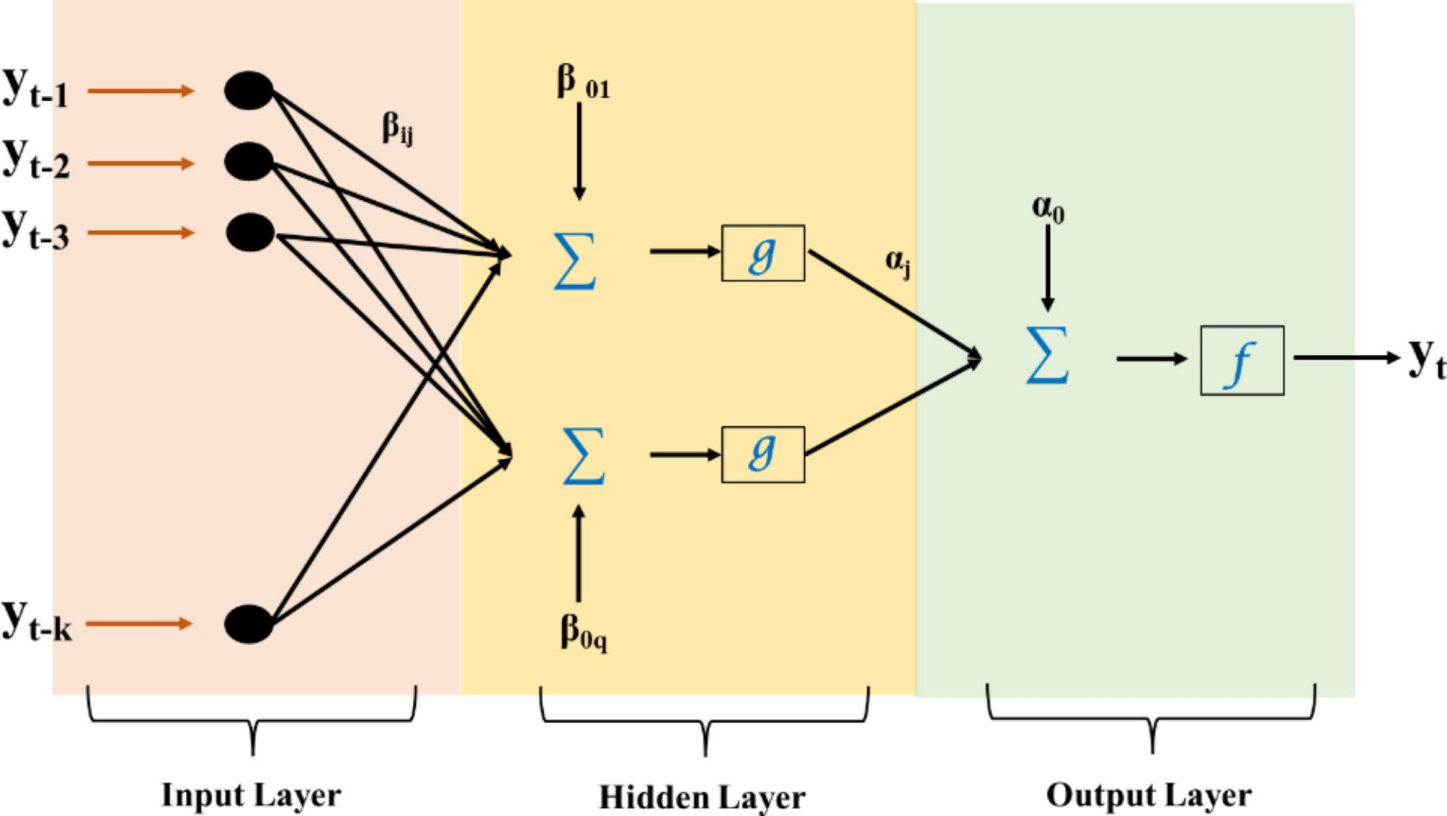
Model architecture of ANN.

 ANN is an ML model inspired by the brain’s structure and function. The architecture of an ANN typically consists of an input layer, one or more hidden layers, and an output layer. The input layer receives and passes the input data to the hidden layers. The hidden layers use weights and biases to transform the input data into a new representation that is more suitable for the task. The output layer produces the final predictions based on the information processed by the hidden layers. The weights and biases are learned during training, allowing the model to make increasingly accurate predictions. The number of hidden layers, the size of the hidden layers, and the activation functions used determine the capacity of the model and its ability to learn complex relationships between inputs and outputs. In Fig. 2, ${y}_{t}$ is considered as a function of its lag values $\:{y}_{t-1},{y}_{t-2},\dots\:,\:{y}_{t-k}$ and respective weights.

1 $$\:{y}_{t}={a}_{0}+\sum\:_{j=1}^{q}{a}_{j}g({\beta\:}_{0j}+\sum\:_{i=1}^{k}{\beta\:}_{ij}{y}_{t-i})+{\epsilon\:}_{t}$$

where $\:{a}_{j}\:(j=\text{0,1},2,\dots\:,q)$ and $\:{\beta\:}_{ij}\:(j=\text{0,1},2,\dots\:,q;i=\text{1,2},.,k)$ are the model’s connection weights; k is the number of nodes in the input layer, and q is the number of nodes in the hidden layer.

### Support vector regression (SVR)

 SVR, developed by Vapnik et al.^39^, is a type of ML model used for regression and time series problems. The SVR model architecture consists of the following components. First, the input features are transformed into a high-dimensional space using a kernel function, such as a radial basis function (RBF) or a polynomial function. The transformed features are used to train a model. The model finds the optimal boundary by maximising the margin between the data points and the boundary. The model parameters are estimated using a cost function penalising deviations from the target values. The model makes predictions for new input data by transforming the features into high-dimensional space and applying the linear regression model. The kernel function and the regularisation parameter (C) are hyperparameters that can be tuned to improve the model’s performance. The main advantage of the SVR model is that it can handle complex non-linear relationships between inputs and outputs using a suitable kernel function.

### Random forest (RF)

**Fig. 3:**
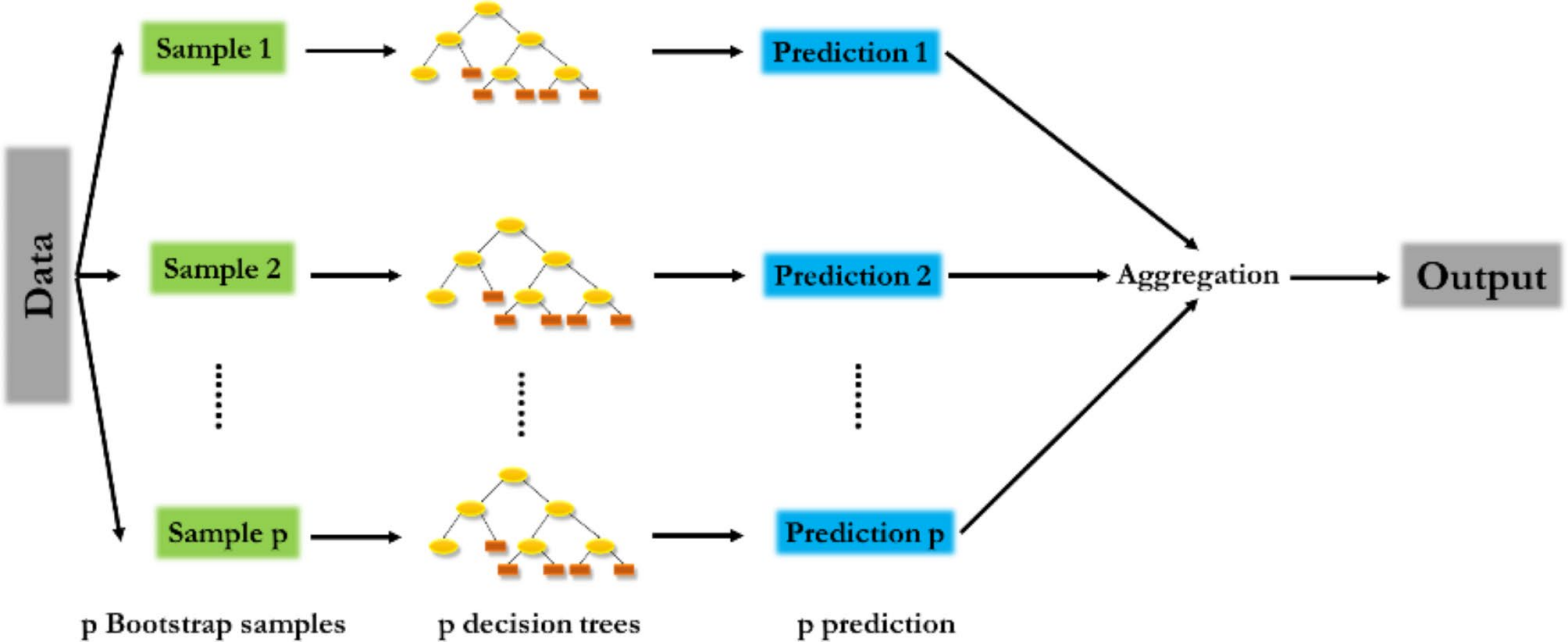
Architecture of RF.

 RF is an ML algorithm based on decision trees. It builds multiple decision trees and combines their predictions to improve the overall accuracy and stability of the model. Many decision trees are trained on randomly selected subsets of the training data. Each decision tree is trained to make predictions for the target variable by recursively splitting the data into subsets based on the values of the input features. The final prediction for a new input sample is the average of the predictions made by all the decision trees. The random selection of features at each split helps to decorrelate the trees and reduce overfitting. The bootstrapped training data samples used to train each tree help reduce the prediction variance. The architecture of the RF model is given in Fig. 3.

### Deep learning (DL) algorithms

 To estimate daily rainfall through current environmental data, three specific algorithms viz., RNN, LSTM, and GRU were selected for the empirical investigation. Consequently, these three DL algorithms were tested and juxtaposed to determine the superior methods for anticipating daily rainfall quantities.

### Recurrent neural network (RNN)

 An RNN is a DL model that processes sequential data. It has a feedback loop that allows information to be passed from one sequence step to the next. The core architecture of an RNN consists of a repeating module with a hidden state, which stores information about the previous time step, and an input-to-hidden layer, which takes the current input and last hidden state as inputs and outputs the current hidden state. The hidden state is then used as input to a hidden-to-output layer, which generates the output for the present time step. The weights of the input-to-hidden and hidden-to-output layers are shared across all time steps, allowing the model to learn patterns in the sequence data. The internal structure of an RNN model can be described using the following equations:

Input Layer: The input layer receives the input sequence denoted as x(t), with t representing the time step.

Hidden Layer: The hidden layer contains the recurrent neuron that computes the hidden state h(t) using the following equation:

2 $$\:\text{h}\left(\text{t}\right)=\text{f}\left({\text{W}}_{hx}\text{*}\text{x}\right(\text{t})+{\text{W}}_{hh}\cdot\:\text{h}(\text{t}-1)+{\text{b}}_{h})$$

where, f is the activation function (e.g., tanh), $\:{\text{W}}_{hx}$ is the weight matrix for the input-to-hidden layer connection,$\:{\text{W}}_{hh}$ is the weight matrix for the hidden-to-hidden layer connection, and $\:{\text{b}}_{h}$ is the bias vector.

Output Layer: The output layer computes the output y(t) using the following equation:

3 $$\:\text{y}\left(\text{t}\right)\:=\:{\text{W}}_{hy}\:\text{*}\:\text{h}\left(\text{t}\right)\:+\:{\text{b}}_{y}$$

where, $\:{\text{W}}_{hy}$ is the weight matrix for the hidden-to-output layer connection, and $\:{\text{b}}_{y}$ is the bias vector.

These equations describe the basic structure of a simple RNN model. More complex RNN models may have additional layers or use different types of activation functions or recurrent neurons. This architecture can be extended with multiple hidden layers to form a deep RNN or multiple hidden states to create an LSTM or GRU.

### Long short-term memory (LSTM) and its variant

**Fig. 4:**
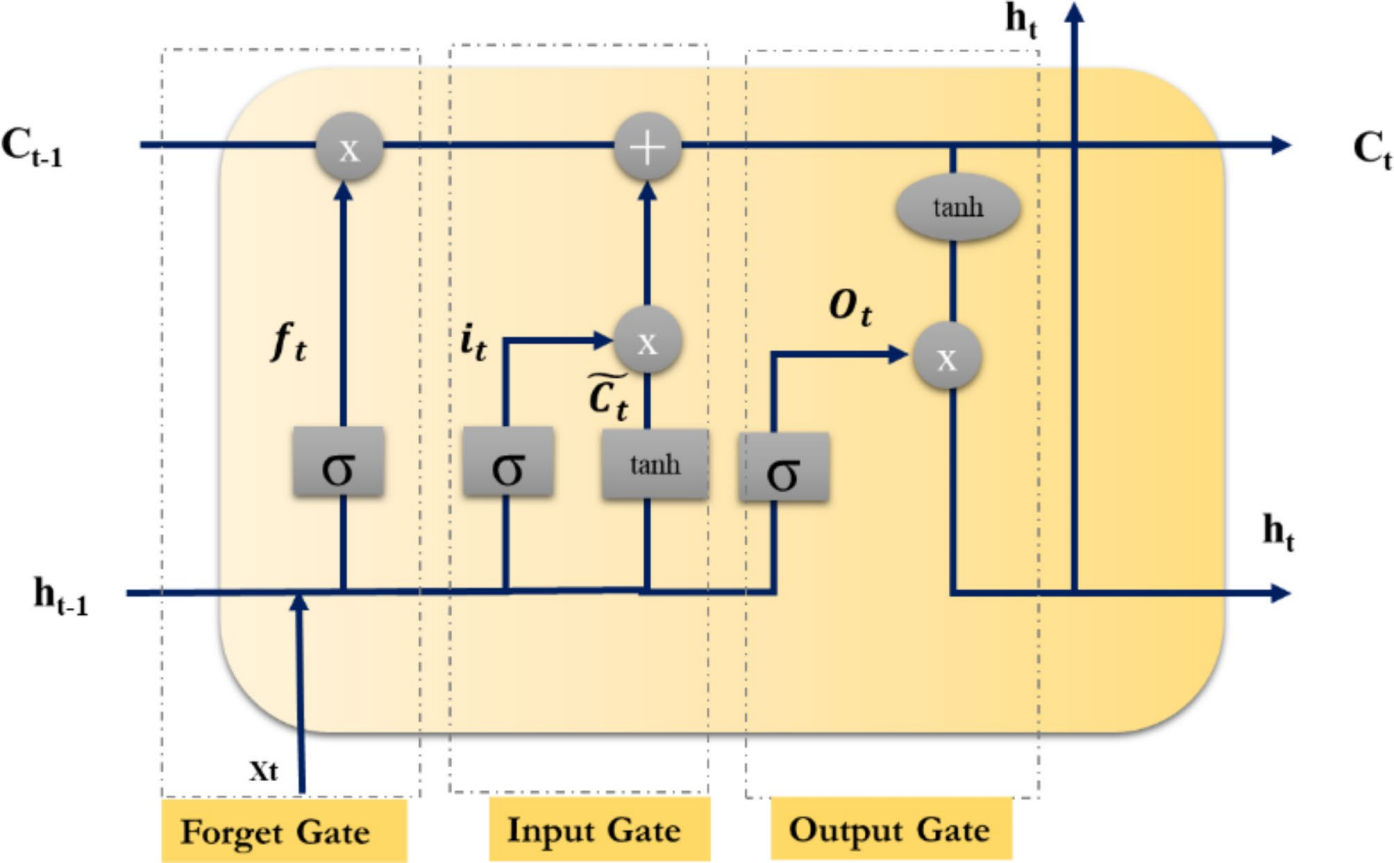
Structure of an LSTM unit.

 LSTM is an RNN type widely used for processing sequential data such as time series, speech signals, and text. It was introduced by^40^ as a solution to the vanishing gradient problem that occurs in traditional RNNs. The LSTM model is an RNN type that can remember previous inputs for a long time. The architecture of an LSTM network consists of memory cells, input gates, forget gates and output gates. The memory cells are responsible for storing information for a prolonged period, whereas the gates control the flow of information in and out of the memory cells. The input gate controls the flow of information into the memory cell, allowing the network to decide what information to retain. The forget gate decides what information should be discarded based on the current input and previous memory state. The output gate determines what information should be outputted based on the current input and previous memory state. The structure of an LSTM unit can be described in the following Fig. 4 and equations:

Input gate: Controls the amount of new information allowed into the cell state. It is calculated using the following equation:

4 $$\:{i}_{t}=\sigma\:\left\{{W}_{i}*\left({h}_{t-1}*{x}_{t}\right)\right\}+{b}_{i}$$

where, $\:{i}_{t}$ is the input gate at time step $\:t$, $\:{W}_{i}$ is the weight matrix,$\:\:{h}_{t-1}$ is the concatenation of the previously hidden state $\:t-1\:$and the current input $\:{x}_{t}$, and $\:{b}_{i}\:$is the bias term. The sigmoid activation function $\:\sigma\:$ ensures that the input gate is between 0 and 1.

Forget gate: Controls the amount of information that will be forgotten from the cell state. It is calculated using the following equation:

5 $$\:{f}_{t}=\sigma\:\left\{{W}_{f}*\left({h}_{t-1}*{x}_{t}\right)\right\}+{b}_{f}$$

where,$\:\:{f}_{t}$ is the forget gate at time step $\:t$, $\:{W}_{f}$ is the weight matrix, and $\:{b}_{f}$ is the bias term.

Cell state: A continuous memory of the model that stores information from the past. It is updated using the following equation:

6 $$\:{C}_{t}={f}_{t}*{C}_{t-1}+{i}_{t}*\stackrel{\sim}{{C}_{t}}$$

where, $\:{C}_{t}$ is the cell state at time step $\:t$, $\:{f}_{t}$ is the forget gate, $\:{i}_{t}$ is the input gate, $\:{C}_{t-1}$ is the previous cell state, and $\:\stackrel{\sim}{{C}_{t}}$ is a candidate cell state calculated as follows:

7 $$\:\stackrel{\sim}{{C}_{t}}=tanh\left\{{W}_{c}*\left({h}_{t-1}*{x}_{t}\right)\right\}+{b}_{c}$$

$\:{W}_{c}$ and $\:{b}_{c}$ are the weight and bias of the candidate’s input.

Output gate: Controls the amount of information output from the cell state to the hidden state. It is calculated using the following equation:

8 $$\:{o}_{t}=\sigma\:\left\{{W}_{o}*\left({h}_{t-1}*{x}_{t}\right)\right\}+{b}_{o}$$

where, $\:{o}_{t}$ is the output gate at time step $\:t$, $\:{W}_{o}$ is the weight matrix, and

$\:{b}_{o}$ is the bias term.

Hidden state: A representation of the current state of the model. It is calculated using the following equation:

9 $$\:{h}_{t}=\:{o}_{t}*tanh\left({C}_{t}\right)$$

where, $\:{h}_{t}$ is the hidden state at time step $\:t$, $\:{o}_{t}$ is the output gate, and $\:{C}_{t}$ is the cell state.

The above equations form the core of an LSTM unit, and multiple units can be stacked together to create a multi-layer LSTM network. A deep LSTM model is a variant of the LSTM model with multiple layers of LSTM units. A bidirectional LSTM processes the input sequences in two ways: in the forward direction (from start to end) and backward order (from end to start).

### Gated recurrent unit (GRU)

 GRU is a modification of the RNN model with two gates: the reset gate and the update gate. These gates help regulate the flow of information and prevent the vanishing gradient problem that can occur in traditional RNNs. The architecture of a GRU is like an LSTM network but with fewer parameters.

### Time series algorithms

 ARIMA and TBATS were chosen to forecast daily rainfall intensity using real-time environmental data. These algorithms were tested and compared to determine the best approach for accurate daily rainfall predictions.

### Autoregressive integrated moving average (ARIMA)

 ARIMA is a popular time series forecasting algorithm to model and predict univariate data. For the ARIMA model, the time series data must be stationary, meaning its mean, variance, and covariance are constant over time. If the data is not stationary, a differencing step is applied to make it stationary. The ARIMA model is selected based on the values of p, d, and q, where p is the number of AR terms, d is the number of differencing steps applied, and q is the number of MA terms. The autocorrelation function (ACF) and partial autocorrelation function (PACF) are used to identify the patterns in the time series data and to determine the number of autoregressive (AR) and moving average (MA) terms to include in the ARIMA model. The model is fitted to the time series data using maximum likelihood estimation or a variation thereof. The model is validated using residual analysis and by comparing the predicted values with the actual values for a portion of the time series data set aside for validation. The ARIMA model predicts the future values of the time series data.

### Exponential smoothing state space model with Box–Cox transformation, ARMA errors, trend and seasonality components (TBATS)

 TBATS is a time series forecasting algorithm for univariate time series data. It is a hybrid algorithm that combines exponential smoothing and ARIMA models, making it suitable for modeling complex time series patterns like seasonality, trend, and irregularity. The algorithm models the time series as a combination of multiple components, including a trend, a seasonal component, and an irregular component. The algorithm estimates the model’s parameters using maximum likelihood estimation and predicts future values by combining the different elements. TBATS has been found to perform well on various time series datasets, including those with multiple seasonal patterns, non-stationary trends, and irregular fluctuations.

### Statistical evaluation

 Generally, it is necessary to assess the performance of a developed prediction model and compare it with other models using specific statistical measures. However, it is crucial to employ multiple statistical indices because different models may yield similar or nearly identical values for a particular index. This similarity makes it challenging to definitively determine which model performs better than the others. Each statistical index evaluates the model’s performance from a single perspective of how well its outputs match the desired values. Therefore, evaluating models across multiple statistical indices is advisable to comprehensively assess each model’s performance and conduct a robust comparative analysis, ultimately identifying the most suitable modeling approach. Two performance metrics were commonly used to evaluate the effectiveness of predictive models: the RMSE, and MAE. RMSE measures the average magnitude of the errors between predicted and actual values. Lower RMSE values indicate better model accuracy, as they signify smaller discrepancies between predicted and observed data points. Similarly, MAE quantifies the average absolute difference between predicted and actual values, with lower values indicating more accurate predictions. The RMSE and MAE can be defined as

$$\:\text{R}\text{M}\text{S}\text{E}=\frac{1}{n}\sqrt{\sum\:_{t=1}^{n}{\left({\text{Y}}_{\text{t}}-\widehat{{\text{Y}}_{\text{t}}}\right)}^{2}}$$

$$\:\text{M}\text{A}\text{E}=\frac{1}{\text{n}}\sum\:_{\text{t}=1}^{\text{n}}\left|{\text{Y}}_{\text{t}}-{\widehat{\text{Y}}}_{\text{t}}\right|$$

where $\:{\text{Y}}_{\text{t}}$ is the actual value, $\:\widehat{{\text{Y}}_{\text{t}}}$ is the fitted value and n is the number of observations.

## Result and discussion

The descriptive statistics of temperature across altitudinal gradients in the North-Western Himalayas reveal significant variability, which is crucial for understanding local climate dynamics and its implications for rainfall patterns. Mean maximum temperatures ranged from 11.65 to 20.21 ℃ across locations L1 to L6, reflecting diverse thermal regimes influenced by altitude (Table 1). These findings are consistent with previous studies emphasizing the role of elevation in temperature variation^41^. The wide range of maximum temperature values, from − 5.70 to 37.60 ℃, reveals the broad climatic spectrum within the study area, indicative of both alpine and lower altitude climates. Similarly, mean minimum temperatures ranged from 2.50 to 7.64 ℃ across the same locations, highlighting cooler conditions at higher elevations compared to lower lying areas (Table 2). This gradient is critical for understanding temperature-dependent processes such as evapotranspiration and precipitation formation^42^. Standard error and skewness values for both maximum and minimum temperatures further elucidate the distribution characteristics, indicating slight asymmetry in temperature data distributions but with relatively low variability, suggesting robustness in measurements^34,43,44^. These temperature statistics serve as a foundational framework for predicting rainfall patterns in the region. Studies have shown that temperature gradients directly influence atmospheric stability, moisture content, and the onset of precipitation events^45–48^. By integrating altitude-specific temperature data into rainfall models, researchers can enhance the accuracy of predictive models tailored to mountainous regions^49^.

**Table 1:** Descriptive statistics of maximum temperature at different altitudinal gradients in the North-Western Himalayas.

Descriptive statistics	Maximum temperature					
	L1	L2	L3	L4	L5	L6
Mean	18.62	16.60	20.02	19.31	11.65	20.21
Standard error	0.07	0.07	0.07	0.07	0.07	0.08
Median	19.96	17.90	21.10	20.70	12.22	21.60
Mode	28.00	25.00	29.00	27.50	21.60	30.50
Standard deviation	8.76	8.35	8.92	8.44	7.96	9.30
Kurtosis	− 1.11	− 1.14	− 1.07	− 1.00	− 1.19	− 1.13
Skewness	− 0.29	− 0.29	− 0.28	− 0.37	− 0.15	− 0.28
Range	41.00	40.20	43.60	40.80	38.70	43.30
Minimum	− 5.70	− 8.00	− 6.60	− 5.10	− 9.60	− 5.70
Maximum	35.30	32.20	37.00	35.70	29.10	37.60

**Table 2:** Descriptive statistics of minimum temperature at different altitudinal gradients in the North-Western Himalayas.

Descriptive statistics	Minimum temperature					
	L1	L2	L3	L4	L5	L6
Mean	6.48	3.04	7.64	6.44	2.50	6.39
Standard error	0.06	0.06	0.06	0.06	0.06	0.06
Median	6.40	3.00	7.40	6.10	2.80	6.20
Mode	0.40	5.00	0.00	0.00	12.40	0.40
Standard deviation	6.95	7.09	7.51	7.12	7.20	7.19
Kurtosis	− 0.98	− 0.57	− 1.11	− 0.94	− 1.08	− 1.05
Skewness	0.01	− 0.07	0.09	0.08	− 0.11	0.12
Range	38.60	64.60	37.00	36.40	37.90	39.10
Minimum	− 15.70	− 18.60	− 11.80	− 13.60	− 19.80	− 15.70
Maximum	22.90	46.00	25.20	22.80	18.10	23.40

The analysis of rainfall patterns across different altitudinal locations (L1 to L6) in the North-Western Himalayas provided valuable insights into the spatial variability and predictive challenges associated with precipitation modeling in mountainous regions. Mean rainfall values ranging from 1.94 to 4.09 mm illustrate the significant diversity in precipitation levels across relatively small geographical distances (Table 3). This variability is further underscored by the wide range of minimum and maximum rainfall values observed at each location, reflecting the complex interplay of topography, atmospheric dynamics, and local climatic conditions. The standard error and skewness values offer additional perspectives on the distributional characteristics of rainfall data. Higher skewness values, such as those observed for locations L2, L3, L4, and L5, indicate asymmetric distributions with a tendency towards higher or lower rainfall extremes, potentially influenced by localized weather phenomena like orographic lifting or convective processes^44,50^. Conversely, lower skewness values suggest a more symmetrical distribution of rainfall, indicative of more moderate and consistent precipitation patterns^51–54^. The application of ML models (RF, SVR, ANN, and KNN) to predict rainfall from meteorological variables reveals varying model performances across different altitudinal gradients. RF and SVR models generally exhibited higher RMSE values during both training and testing phases, suggesting challenges in capturing the complex nonlinear relationships between predictors and rainfall outcomes in mountainous terrain. These findings align with previous studies emphasizing the sensitivity of statistical models to spatial heterogeneity and the need for robust validation strategies in mountainous regions^55–57^. In contrast, ANN and KNN models demonstrated relatively lower RMSE values, indicating their potential for better capturing spatial variability and nonlinearity in rainfall patterns across diverse altitudes. The superior performance of these models may stem from their ability to learn complex patterns and relationships inherent in meteorological data, including altitude-dependent factors such as orographic effects and microclimatic variations^58,59^. These findings highlight the importance of integrating altitude-specific meteorological data and employing advanced modeling techniques to enhance the accuracy of rainfall forecasts in mountainous regions. Future research directions could include refining model inputs by incorporating additional environmental variables (e.g., terrain characteristics, vegetation cover) and exploring ensemble modeling approaches to mitigate uncertainties associated with individual model performances^60–64^. Furthermore, incorporating high-resolution satellite data and ground-based observations could further improve the spatial representation of rainfall patterns and support more robust model validations in complex terrain settings.

**Table 3:** Descriptive statistics of rainfall at different altitudinal gradients in the North-Western Himalayas.

Descriptive statistics	Rainfall					
	L1	L2	L3	L4	L5	L6
Mean	2.98	3.51	1.94	3.32	4.09	2.97
Standard Error	0.07	0.07	0.05	0.09	0.08	0.06
Median	0.00	0.00	0.00	0.00	0.00	0.00
Mode	0.00	0.00	0.00	0.00	0.00	0.00
Standard Deviation	8.26	8.70	6.30	10.37	10.13	7.65
Kurtosis	44.80	33.66	56.41	53.54	49.59	21.75
Skewness	5.46	4.77	6.18	6.02	5.61	4.14
Range	149.50	138.90	130.30	206.00	189.20	85.00
Minimum	0.00	-5.50	0.00	0.00	-0.20	0.00
Maximum	149.50	133.40	130.30	206.00	189.00	85.00

**Fig. 5:**
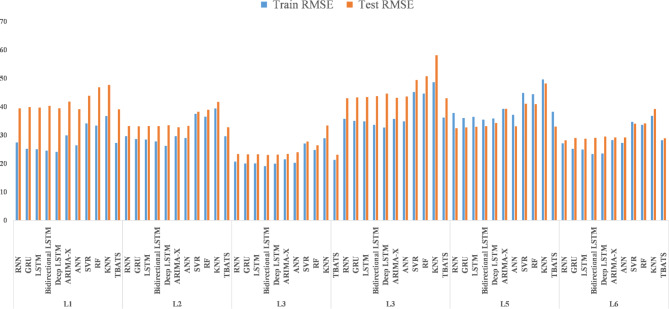
Bar plot of RMSE across altitudinal gradient.

The application of ML, DL, and time series modeling techniques to predict rainfall from meteorological variables across altitudinal gradients in the North-western Himalayas offers valuable insights into model performance and predictive accuracy (Table 4). ML models such as RF, SVR, ANN, and KNN exhibited varying levels of effectiveness as indicated by their train and test RMSE values across altitudinal levels (L1 to L6) (Fig. 5). These differences underscored the sensitivity of these models to spatial variations in climatic conditions and the complex interactions between meteorological predictors and rainfall patterns. DL models, including LSTM, bi-directional LSTM, deep LSTM, GRU, and RNN, also demonstrated varying RMSE values across altitudinal gradients. These models leverage sequential dependencies in data and have shown promise in capturing temporal patterns and non-linear relationships in climatic variables, which are crucial for accurate rainfall prediction in dynamic mountainous environments^40,65^. Moreover, time series modeling approaches such as ARIMA and TBATS provided insights into the temporal variability of rainfall across altitudes. These models exhibited varying train and test RMSE values, reflecting their ability to capture both short-term fluctuations and long-term trends in rainfall data^56,66–68^. The observed differences in RMSE values across these modeling techniques highlight the importance of selecting appropriate methodologies that account for the complex spatial and temporal dynamics inherent in mountainous regions. The higher RMSE values in some models indicate challenges in accurately capturing local-scale variations and extreme weather events, which are critical for effective water resource management and disaster preparedness in mountain ecosystems^69–71^. Integrating diverse modeling approaches and leveraging advanced statistical techniques enhance our understanding of rainfall variability in mountainous areas.

**Table 4:** Performance metrics of predictive models at different altitudinal gradients in the North-Western Himalayas.

Models	L1				L2				L3			
	Train RMSE	Test RMSE	Train MAE	Test MAE	Train RMSE	Test RMSE	Train MAE	Test MAE	Train RMSE	Test RMSE	Train MAE	Test MAE
LSTM	26.4051	38.2848	18.3032	22.272	28.6268	32.6531	18.6174	20.2858	20.9055	22.4477	14.0341	14.8548
RNN	26.4314	38.4852	17.9959	22.048	34.4668	36.1414	28.5762	28.7417	23.2679	24.2392	18.0615	18.1207
Bidirectional LSTM	28.5082	39.0882	20.9806	24.3876	28.4072	32.4625	18.7237	20.3433	20.738	22.2265	13.966	14.6357
Deep LSTM	29.2431	39.3847	22.2205	25.5191	30.2622	33.0527	22.1146	23.2161	22.7085	23.8022	17.0902	17.3248
GRU	29.5688	39.4416	22.7908	25.8663	31.0385	33.4672	23.8292	24.8423	22.0524	23.0137	14.7668	15.453
TBATS	30.4407	39.9763	23.7599	26.9549	33.7797	35.5372	26.5045	26.1142	23.6468	24.5349	17.885	17.2726
ANN	29.5231	40.6845	21.3692	23.5784	31.4014	37.3677	23.3202	27.4649	22.0548	24.0753	15.5666	15.8668
KNN	39.137	44.8777	26.0212	26.8164	41.1062	42.5591	29.3555	30.6486	29.0738	29.6286	19.4788	20.0675
SVR	37.0156	46.6131	25.3716	30.5223	40.2145	42.0613	29.3366	30.4928	28.7573	29.8899	19.6855	20.3037
RF	36.5499	48.5559	25.5494	31.5407	42.4912	43.1474	29.9277	29.323	30.6046	33.4445	19.9559	21.6242
ARIMA-X	33.3473	48.9682	24.8915	40.2202	33.7015	35.8058	27.2914	28.2475	23.2583	24.5984	17.7394	18.571

Models	L4				L5				L6			
	Train RMSE	Test RMSE	Train MAE	Test MAE	Train RMSE	Test RMSE	Train MAE	Test MAE	Train RMSE	Test RMSE	Train MAE	Test MAE
LSTM	37.6824	41.882	25.7388	23.2889	36.9767	32.2304	22.3422	21.5748	27.0726	27.1765	19.3609	20.128
RNN	34.9175	42.6456	21.6094	22.2904	36.6707	32.2461	22.3529	21.7823	27.7091	28.0215	19.5725	20.5426
Bidirectional LSTM	34.9437	42.6471	21.2045	21.8476	36.815	34.6105	25.4816	25.6676	26.5043	26.9585	18.4087	19.5685
Deep LSTM	36.629	44.0575	25.7735	26.5994	38.4883	35.9311	26.3701	26.5954	28.9267	29.2136	22.0771	22.8641
GRU	36.6263	44.1257	25.4547	26.1814	41.3881	35.9506	30.7579	27.3958	28.2329	28.6365	21.3745	22.0169
TBATS	39.4747	45.1084	30.271	29.8734	39.0508	37.0425	27.7653	28.1527	30.4776	30.9879	23.0755	24.0622
ANN	37.902	45.2663	28.3823	29.2586	39.0998	37.0643	27.8667	28.2929	26.633	30.0485	19.4238	20.9501
KNN	45.3611	51.0009	32.2122	38.5397	45.1007	40.61	31.9854	27.5973	37.01	37.7642	25.2801	27.1359
SVR	48.4088	52.6685	32.5023	33.006	47.412	44.517	32.5436	30.7088	36.3563	37.1531	25.3094	26.9675
RF	47.8864	53.3362	32.6218	32.8244	47.9481	45.5619	32.3674	31.2958	38.7174	37.8615	25.8642	25.9618
ARIMA-X	51.0779	58.2526	33.3209	35.5394	51.7974	53.9499	33.4704	34.7775	31.444	30.9085	24.5291	24.6846

**Fig. 6:**
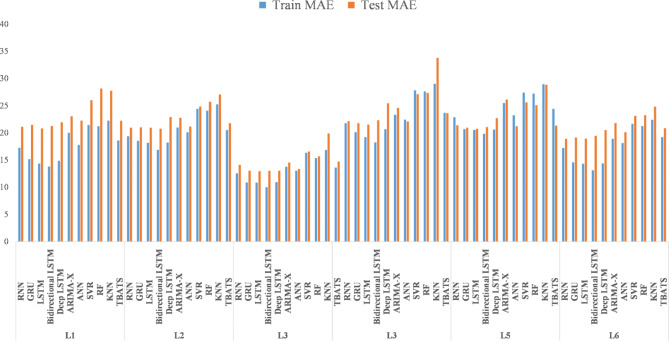
Bar plot of MAE across altitudinal gradient.

The DL models, including LSTM, RNN, Bidirectional LSTM, deep LSTM, and GRU, exhibited varying degrees of effectiveness across the study locations. LSTM consistently demonstrated robust performance, achieving lower RMSE and MAE compared to other DL models in several instances^30^. For example, at Location 1, LSTM achieved a training RMSE of 26.4051 and a testing RMSE of 38.2848, indicating its proficiency in capturing the complex relationships between temperature variables and rainfall patterns. Bidirectional LSTM also showed competitive performance, particularly noteworthy for its lower MAE scores across multiple locations. However, it displayed marginally higher RMSE values compared to LSTM, suggesting potential variability in predictive accuracy across different evaluation metrics. On the other hand, Deep LSTM and GRU, while generally performing adequately, exhibited higher RMSE and MAE values compared to LSTM and Bidirectional LSTM (Fig. 6). This observation suggests that these models might have struggled more with capturing the intricate nuances of temperature-rainfall relationships specific to the north-western Himalayan region. In contrast, traditional ML models such as ANN, KNN, SVR, RF, and the time series approach ARIMA consistently demonstrated higher RMSE and MAE values across all locations^72^. Specifically, KNN, SVR, and RF exhibited notably higher error metrics, highlighting their limitations in accurately capturing the non-linear dependencies inherent in rainfall prediction tasks compared to DL models.

**Fig. 7:**
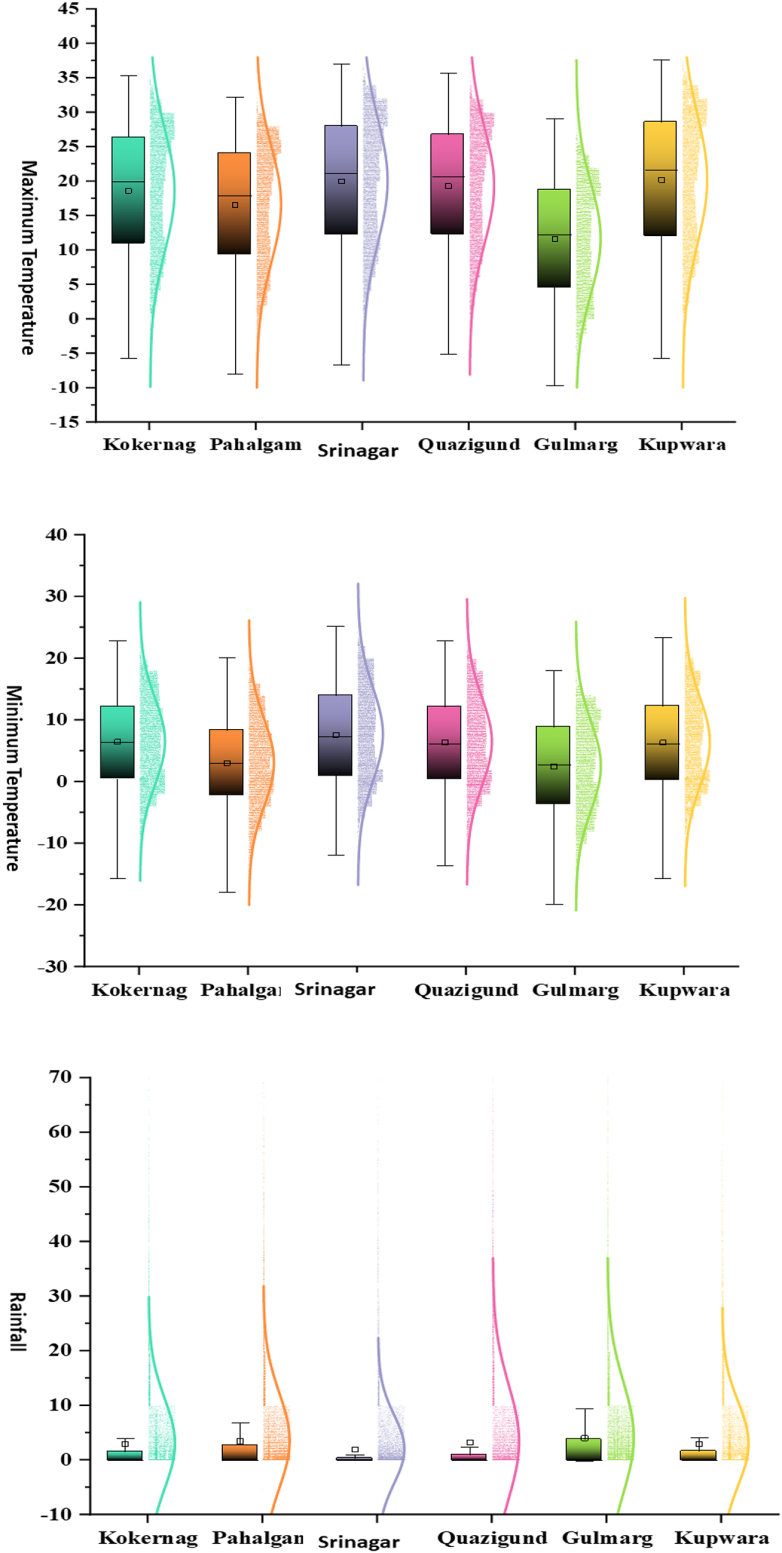
Distribution of weather variables across altitudinal gradient.

**Fig. 8:**
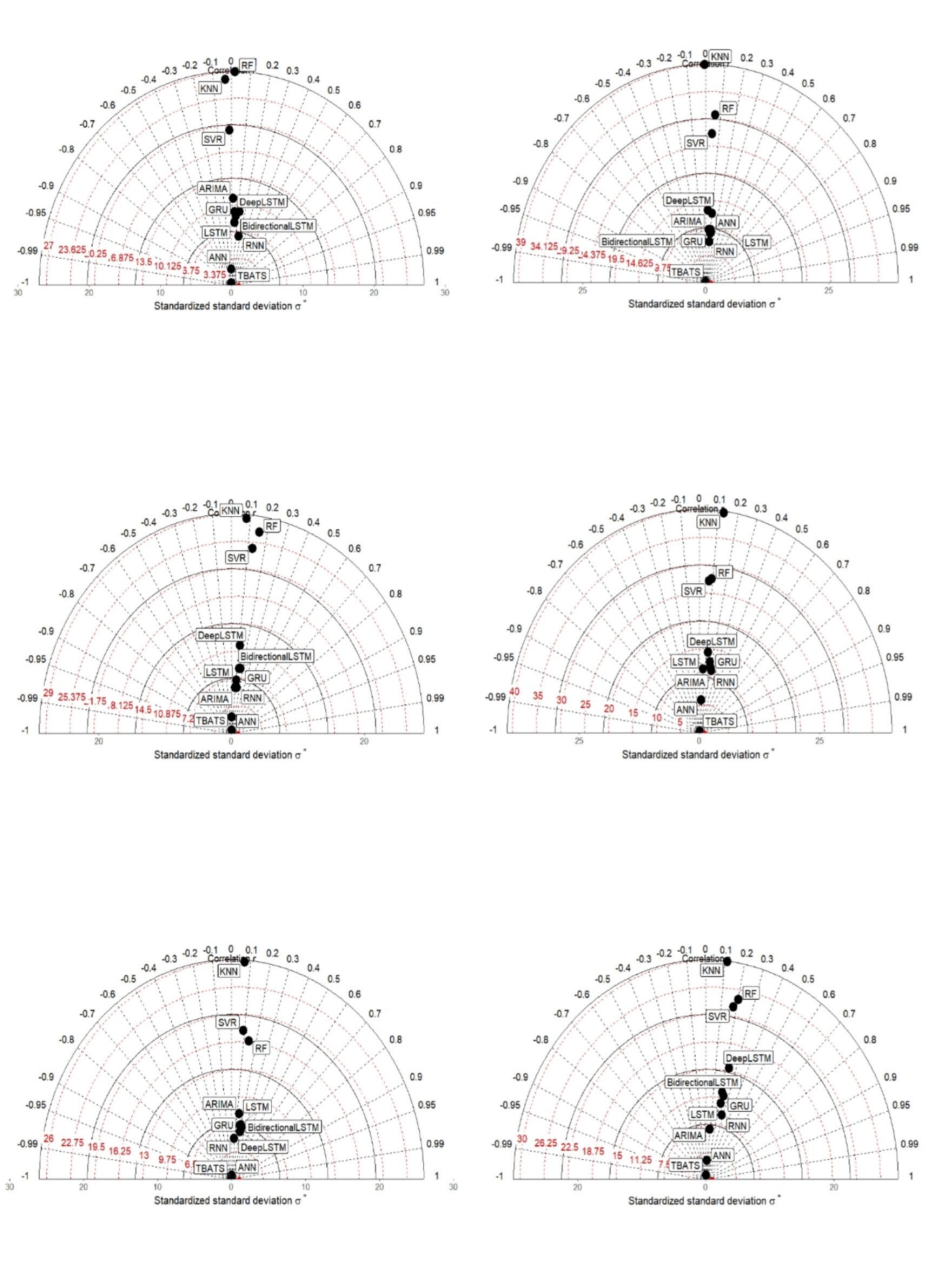
Taylor diagram of altitude wise comparing accuracy of models.

The DL models consistently outperformed both ML and time series models across all six locations examined (Fig. 7). Specifically, LSTM and Bidirectional LSTM emerged as the top-performing models, achieving the lowest RMSE and MAE scores for locations L1 through L6, respectively. Meanwhile, RNN, LSTM, Bidirectional LSTM, and Deep LSTM were identified as superior models based on their performance in terms of test MAE across the same locations. Overall, the DL algorithms demonstrated superior accuracy, with Bi-directional LSTM showing the highest effectiveness, followed by LSTM, RNN, Deep LSTM, and GRU in descending order. In contrast, the ML models performed relatively better than traditional time series methods, with ANN leading, followed by KNN, SVR, and RF. Time series models, represented by TBATS and ARIMA, ranked lowest in accuracy (Fig. 8). The preference for DL models in predicting rainfall patterns in the North-Western Himalayas can be attributed to several factors. Firstly, the complex and non-linear nature of the data, influenced by altitude variations, topography, and atmospheric stability, poses challenges for ML and time series models in accurately capturing these relationships^73^. DL models, such as LSTM and Bidirectional LSTM, are specifically designed to handle such complexities by automatically learning intricate data patterns^27,72^. This capability reduces the need for manual feature engineering and enhances prediction accuracy by effectively managing noise inherent in rainfall and temperature data. DL models benefit from the availability of large historical datasets in the region, which are essential for training these models effectively. The extensive data enable DL models to generalize well and remain robust against temporal variations in the data, thereby improving overall prediction accuracy. This study makes a significant contribution to overcoming challenges in agricultural planning and climate adaptation by applying advanced Machine Learning (ML) and Deep Learning (DL) techniques to rainfall prediction. As traditional forecasting methods struggle with the increasing unpredictability of climate, this research enhances forecast accuracy through sophisticated algorithms, allowing for more informed decisions by farmers and agricultural planners. By integrating complex climatic data and assessing model effectiveness in the North-Western Himalayas, the study provides crucial insights into improving predictive precision and managing varied environmental conditions. This advancement supports better agricultural practices, infrastructure development, and overall food security in the face of climate variability.

The use of advanced Machine Learning (ML) and Deep Learning (DL) techniques for rainfall prediction, as outlined in this study, represents a significant advancement in meteorological forecasting with important implications for engineers and stakeholders, especially in semi-arid regions. Techniques such as Random Forest (RF), Support Vector Regression (SVR), Long Short-Term Memory (LSTM), and Gated Recurrent Units (GRU) offer notable improvements in rainfall prediction accuracy. This enhanced precision is crucial for effective planning and management in areas dependent on seasonal rains. However, applying these techniques in practice brings several considerations. A key issue is balancing the accuracy of the models with their complexity. While these advanced methods can deliver highly accurate forecasts, they also introduce increased computational demands and complexity. Engineers and stakeholders must assess whether the benefits of improved accuracy justify the challenges associated with implementing and maintaining these sophisticated systems. The models require significant computational power and specialized expertise, which might be a hurdle for regions with limited technological resources. Proper integration of these advanced models into existing forecasting systems is necessary to ensure that the accuracy improvements justify the operational demands. Another important consideration is the dependency on extensive and high-quality datasets. These advanced models perform best with large amounts of data, which may not always be available, especially in regions with sparse meteorological information. This points to a need for better data collection infrastructure. Engineers should focus on enhancing data acquisition systems and developing strategies to handle limited data to fully utilize these forecasting techniques.

The adaptability of these methodologies to other meteorological variables like humidity, wind speed, and solar radiation is also significant. While the principles behind these methods are versatile, each climatic variable has unique challenges that may require specialized approaches. Although the methods used for rainfall prediction can be extended to other variables, engineers need to tailor the models to each specific context to ensure accuracy and effectiveness. Operational integration is another practical challenge. Incorporating these advanced models into existing systems for agricultural planning, disaster management, and infrastructure development requires careful consideration. Stakeholders must address how to integrate these models into practical decision-making processes, which includes not only technical adaptation but also training and capacity building for users. Ensuring that personnel are properly trained to use these advanced tools is essential for maximizing their benefits. The benefits of improved rainfall forecasting are considerable. Enhanced prediction accuracy enables better agricultural planning, such as more precise irrigation scheduling and effective water resource management. It also supports infrastructure design and disaster preparedness by helping engineers create infrastructure that can withstand extreme weather events. For policymakers, advanced forecasting techniques provide a foundation for developing data-driven policies that promote sustainable resource management and enhance climate resilience. In summary, while advanced techniques for rainfall prediction offer considerable advantages, they also require careful management of model complexity, data needs, and operational integration. Addressing these challenges effectively can lead to significant improvements in decision-making, infrastructure planning, and policy development, ultimately supporting more resilient and sustainable practices in meteorology and related fields. The model parameters were presented in Table 2S.

## Conclusions

In conclusion, the comprehensive analysis of temperature and rainfall patterns across altitudinal gradients in the North-Western Himalayas portrays the significant variability and complexity inherent in mountainous climatic systems. The study revealed diverse thermal regimes influenced by altitude, with mean maximum temperatures ranging from 11.65 to 20.21 ℃ and mean minimum temperatures from 2.50 to 7.64 ℃ across different locations. Altitude is a critical factor shaping temperature variations in the region. The wide range of temperature values reflects both alpine and lower altitude climates, crucial for understanding local climate dynamics, evapotranspiration processes, and precipitation formation. Statistical analyses, including standard error and skewness, further elucidate the distribution characteristics of temperature data, emphasizing robustness in measurements despite slight asymmetry. This foundational framework of temperature statistics not only enhances our understanding of regional climate but also serves as a crucial basis for predicting rainfall patterns. Integrating altitude-specific temperature data into predictive models improves the accuracy of rainfall forecasts by accounting for temperature gradients that influence atmospheric stability, moisture content, and precipitation onset. Advanced modeling techniques such as ML, DL, and time series analysis provided deeper insights into rainfall variability across diverse altitudinal gradients. DL models, particularly LSTM and Bidirectional LSTM, demonstrated superior performance in capturing complex climatic relationships compared to traditional ML and time series methods. Their ability to handle non-linear data dynamics and leverage extensive historical datasets underscores their effectiveness in predicting rainfall patterns in dynamic mountainous environments. Moving forward, continued research efforts should focus on refining model inputs by incorporating additional environmental variables and exploring ensemble modeling approaches to further enhance prediction accuracy. High-resolution satellite data and ground-based observations will play pivotal roles in improving spatial representation and validating models in complex terrain settings. By advancing our understanding and predictive capabilities, we can better manage water resources and mitigate risks associated with climate variability in mountain ecosystems. In real-world applications for rainfall monitoring and warning systems, standalone methods such as statistical, physical, and data-driven models offer distinct advantages but also face notable limitations. Statistical models, like ARIMA, are straightforward and computationally efficient, making them practical for immediate use. However, their tendency to assume linear relationships and their limited flexibility can lead to suboptimal performance when faced with sudden climatic changes or complex weather patterns. Physical models, which simulate atmospheric and hydrological processes, can provide in-depth forecasts by considering intricate variable interactions. Despite their accuracy in well-defined conditions, these models are often hindered by high computational demands and complexity, making them less feasible for real-time applications in regions with limited resources. Additionally, physical models may also encounter local optima if not precisely calibrated for specific regional conditions. Data-driven models, such as machine learning and deep learning techniques, are adept at identifying complex, non-linear patterns and can be adapted to various datasets. While they have the potential for high precision, their effectiveness depends on access to extensive and high-quality data and significant computational power. These models are also prone to overfitting, where they excel with historical data but may underperform with new or different data, and their intricate nature often results in lower interpretability. Overall, while advanced data-driven methods offer substantial improvements in rainfall forecasting, their practical implementation must carefully address data quality, computational requirements, and ongoing maintenance to ensure reliable and actionable predictions.

## Electronic supplementary material

Below is the link to the electronic supplementary material.

Supplementary Material 1

## Data Availability

The datasets used and/or analyzed during the current study are available from the corresponding author on reasonable request.
