# Supplementary material for: Predicting rainfall using machine learning, deep learning, and time series models across an altitudinal gradient in the North-Western Himalayas
Source: Sci Rep. 2024 Nov 13;14:27876. doi: 10.1038/s41598-024-77687-x (PMC11561348; doi:10.1038/s41598-024-77687-x)
Supplement: Supplementary file 1 — Supplementary Material 1 [file 41598_2024_77687_MOESM1_ESM.docx]

| **Machine learning models** | | | | | | | | | | | |
| --- | --- | --- | --- | --- | --- | --- | --- | --- | --- | --- | --- |
| **Locations** | **ANN** | | | | **RF** | | | | **SVM** | | |
| **L1** | Lags of independent variables | Hidden Layers | Learning Rate | sigma^2 | Number of trees | No. of variables tried at each split | Mean of squared residuals | % Var explained | SVM-Type | SVM-Kernel | Number of Support Vectors |
| **L2** | 10 | 13 | 0.0097 | 0.0029 | 500 | 19 | 0.000241111 | 93.02 | eps-regression | radial | 582 |
| **L3** | 10 | 12 | 0.0083 | 0.01094 | 500 | 18 | 0.000647311 | 95.35 | eps-regression | radial | 611 |
| **L4** | 9 | 12 | 0.0099 | 0.008042 | 500 | 18 | 0.000649312 | 93.54 | eps-regression | radial | 554 |
| **L5** | 12 | 11 | 0.0096 | 0.002973 | 500 | 20 | 0.000198448 | 94.25 | eps-regression | radial | 553 |
| **L6** | 11 | 15 | 0.0088 | 0.006004 | 500 | 22 | 0.000731267 | 91.35 | eps-regression | radial | 540 |

**Table 1S:** Model parameters and model information

| **Deep Learning** | | | | | | | | | | | | | | | | | | | |
| --- | --- | --- | --- | --- | --- | --- | --- | --- | --- | --- | --- | --- | --- | --- | --- | --- | --- | --- | --- |
| **Locations** | LSTM | | | | **Bidirectional LSTM** | | | | **Deep LSTM** | | | **GRU** | | | | **RNN** | | | |
| **L1** | Lag of Dependent variables | Lags of independent variables | | LSTM Layers | Lag of Dependent variables | Lags of independent variables | | Bi-LSTM Layers | Lag of Dependent variables | | Lags of independent variables | Lag of Dependent variables | | Lags of independent variables | GRU Layers | Lag of Dependent variables | | Lags of independent variables | RNN Layers |
| **L2** | 21 | 10 | | 35 | 21 | 10 | | 33 | 21 | | 10 | 21 | | 10 | 39 | 21 | | 10 | 41 |
| **L3** | 20 | 10 | | 29 | 20 | 10 | | 21 | 20 | | 10 | 20 | | 10 | 33 | 20 | | 10 | 39 |
| **L4** | 20 | 9 | | 27 | 20 | 9 | | 26 | 20 | | 9 | 20 | | 9 | 36 | 20 | | 9 | 43 |
| **L5** | 22 | 12 | | 31 | 22 | 12 | | 30 | 22 | | 12 | 22 | | 12 | 34 | 22 | | 12 | 28 |
| **L6** | 24 | 11 | | 30 | 24 | 11 | | 33 | 24 | | 11 | 24 | | 11 | 33 | 24 | | 11 | 35 |
| **Time Series modelling** | | | | | | | | | | | | | | | | | | | |
| **Locations** | | | **ARIMA** | | | | | | | | | | **TBATS** | | | | | | |
|  |  |  | **sigma^2 estimated** | | | | **log likelihood** | | | **aic** | | | **Sigma** | | | | **AIC** | | |
| L1 | | | 0.003611 | | | | 2300.8 | | | -4551.61 | | | 0.0578155 | | | | 2835.556 | | |
| L2 | | | 0.01106 | | | | 1377.02 | | | -2702.05 | | | 0.1157039 | | | | 5143.185 | | |
| L3 | | | 0.008302 | | | | 1614.42 | | | -3170.84 | | | 0.09907659 | | | | 4630.288 | | |
| L4 | | | 0.004012 | | | | 2213.92 | | | -4379.83 | | | 0.05814985 | | | | 2854.62 | | |
| L5 | | | 0.007791 | | | | 1665.71 | | | -3279.43 | | | 0.08717039 | | | | 4211.026 | | |
| L6 | | | 0.009172 | | | | 1532.05 | | | -3008.11 | | | 0.0845677 | | | | 4326.677 | | |

**Table 2S:** Location and model wise model accuracy

| Model | L1 | | | | L2 | | | | L3 | | | |
| --- | --- | --- | --- | --- | --- | --- | --- | --- | --- | --- | --- | --- |
|  | Train Bias | Test Bias | Train R2 | Test R2 | Train Bias | Test Bias | Train R2 | Test R2 | Train Bias | Test Bias | Train R2 | Test R2 |
| ANN | 17.32 | 16.88 | 0.16 | 0.10 | 18.81 | 24.06 | 0.16 | 0.10 | 12.45 | 12.77 | 0.16 | 0.10 |
| ARIMA-X | 20.67 | 38.37 | 0.15 | 0.15 | 23.08 | 22.34 | 0.25 | 0.19 | 15.82 | 14.34 | 0.22 | 0.15 |
| Bidirectional LSTM | 17.50 | 20.01 | 0.47 | 0.48 | 18.27 | 19.03 | 0.54 | 0.54 | 14.59 | 15.09 | 0.42 | 0.49 |
| Deep LSTM | 18.63 | 20.67 | 0.47 | 0.46 | 19.30 | 20.85 | 0.26 | 0.27 | 11.07 | 11.99 | 0.46 | 0.49 |
| GRU | 19.48 | 21.93 | 0.49 | 0.51 | 26.32 | 24.94 | 0.50 | 0.49 | 15.93 | 16.34 | 0.45 | 0.50 |
| KNN | 17.52 | 16.87 | 0.19 | 0.14 | 21.22 | 21.22 | 0.17 | 0.10 | 13.54 | 13.95 | 0.13 | 0.11 |
| LSTM | 14.64 | 15.35 | 0.97 | 0.95 | 13.08 | 13.59 | 0.95 | 0.94 | 11.03 | 11.29 | 0.94 | 0.94 |
| RF | 18.45 | 19.74 | 0.12 | 0.11 | 22.32 | 23.03 | 0.09 | 0.09 | 13.85 | 14.21 | 0.14 | 0.15 |
| RNN | 14.02 | 15.12 | 0.97 | 0.97 | 12.68 | 13.12 | 0.95 | 0.94 | 11.73 | 11.93 | 0.94 | 0.94 |
| SVR | 17.93 | 21.09 | 0.10 | 0.07 | 21.47 | 22.46 | 0.18 | 0.17 | 13.31 | 14.45 | 0.13 | 0.14 |
| TBATS | 21.21 | 21.61 | 0.17 | 0.11 | 24.47 | 27.09 | 0.14 | 0.10 | 14.86 | 14.82 | 0.12 | 0.10 |
| Model | L4 | | | | L5 | | | | L6 | | | |
|  | Train Bias | Test Bias | Train R2 | Test R2 | Train Bias | Test Bias | Train R2 | Test R2 | Train Bias | Test Bias | Train R2 | Test R2 |
| ANN | 19.32 | 17.63 | 0.17 | 0.14 | 19.63 | 22.64 | 0.28 | 0.19 | 15.83 | 16.70 | 0.15 | 0.13 |
| ARIMA-X | 23.90 | 35.78 | 0.07 | 0.11 | 24.80 | 19.69 | 0.24 | 0.19 | 19.32 | 21.30 | 0.40 | 0.31 |
| Bidirectional LSTM | 24.26 | 25.48 | 0.49 | 0.60 | 22.89 | 24.14 | 0.69 | 0.66 | 18.70 | 19.54 | 0.45 | 0.40 |
| Deep LSTM | 20.72 | 22.94 | 0.47 | 0.53 | 22.25 | 24.81 | 0.72 | 0.70 | 17.75 | 18.01 | 0.63 | 0.61 |
| GRU | 20.80 | 21.04 | 0.56 | 0.65 | 20.46 | 22.95 | 0.70 | 0.67 | 15.58 | 15.77 | 0.67 | 0.65 |
| KNN | 20.72 | 22.12 | 0.14 | 0.12 | 21.51 | 23.29 | 0.30 | 0.11 | 16.35 | 18.37 | 0.21 | 0.10 |
| LSTM | 13.66 | 14.22 | 0.98 | 0.98 | 14.97 | 17.03 | 0.95 | 0.95 | 14.07 | 15.57 | 0.94 | 0.94 |
| RF | 22.02 | 22.55 | 0.11 | 0.10 | 22.90 | 22.55 | 0.34 | 0.26 | 17.67 | 18.36 | 0.24 | 0.25 |
| RNN | 14.42 | 14.71 | 0.98 | 0.99 | 14.87 | 16.97 | 0.96 | 0.96 | 15.64 | 17.01 | 0.94 | 0.96 |
| SVR | 21.76 | 23.63 | 0.10 | 0.10 | 21.93 | 22.37 | 0.31 | 0.24 | 17.15 | 18.79 | 0.22 | 0.23 |
| TBATS | 26.06 | 23.69 | 0.10 | 0.10 | 26.48 | 25.00 | 0.18 | 0.10 | 21.84 | 22.82 | 0.14 | 0.10 |
